# HPV positivity status in males is related to the acquisition of HPV infection in females in heterosexual couples

**DOI:** 10.1007/s10096-023-04722-6

**Published:** 2024-01-04

**Authors:** Yuxuan Huang, Yafang Kang, Ye Li, Liangzhi Cai, Qibin Wu, Dabin Liu, Xiaodan Mao, Leyi Huang, Kelvin Stefan Osafo, Yan Zhang, Shuxia Xu, Binhua Dong, Pengming Sun

**Affiliations:** 1 Laboratory of Gynecologic Oncology, Fujian Maternity and Child Health Hospital, College of Clinical Medicine for Obstetrics & Gynecology and Pediatrics, Fujian Medical University, 18 Daoshan Road, Fuzhou, 350001 Fujian People’s Republic of China; 2 Fujian Key Laboratory of Women and Children’s Critical Diseases Research, Fujian Maternity and Child Health Hospital (Fujian Women and Children’s Hospital), Fuzhou, 350001 Fujian People’s Republic of China; 3 Fujian Clinical Research Center for Gynecological Oncology, Fujian Maternity and Child Health Hospital (Fujian Obstetrics and Gynecology Hospital), Fuzhou, 350001 Fujian People’s Republic of China; 4 Department of Gynecology, Fujian Maternity and Child Health Hospital, College of Clinical Medicine for Obstetrics & Gynecology and Pediatrics, Fujian Medical University, Fuzhou, 350001 Fujian People’s Republic of China; 5 The State Key Laboratory of Molecular Vaccinology and Molecular Diagnostics, National Institute of Diagnostics and Vaccine Development in Infectious Diseases, School of Public Health, Xiamen University, Xiamen, 361102 Fujian People’s Republic of China; 6 Department of Pathology, Fujian Maternity and Child Health Hospital, College of Clinical Medicine for Obstetrics & Gynecology and Pediatrics, Fujian Medical University, Fuzhou, 350001 Fujian People’s Republic of China

**Keywords:** Human papillomavirus, Heterosexual couples, Prevalence, Sexually transmitted infection

## Abstract

**Purpose:**

Few studies have focused on the impact of human papillomavirus (HPV) positivity in male partners on female HPV infection and cervical lesions. The purpose of this study was to evaluate the impact of the HPV infection status of husbands on wives’ cervical HPV infection and lesions.

**Methods:**

We surveyed 251 monogamous couples who attended the outpatient department of Fujian Maternity and Child Health Hospital from 2013 to 2021. HPV type analysis was performed on exfoliated cells of the females’ cervix and males’ urethra by the PCR-reverse dot blot method. We analyzed the prevalence and consistency of HPV types in 251 couples. Subsequently, the risk of HPV infection in females with HPV-positive male partners was analyzed. SPSS version 26 (IBM, Chicago, USA) was used for statistical analysis.

**Results:**

In 251 couples, the most commonly detected high-risk HPV (HR-HPV) genotypes were 52, 51, 16, and 58 for males and 16, 52, 18, and 58 for females. Wives with HPV-positive husbands had higher infection rates for most HR-HPV genotypes. HR-HPV positivity in husbands was a risk factor for the development of cervical lesions in wives (OR = 2.250, *P* = 0.014). Both single-type (OR = 2.085, *P* = 0.040) and multiple-type (OR = 2.751, *P* = 0.036) infection in husbands will contributed to an increased risk of non-HR-HPV infection and cervical lesions in wives.

**Conclusion:**

Husbands’ HPV positivity increases the burden of non-HR-HPV infection and increases the risk of cervical lesions developing in wives. It is hoped to provide a reference value for cervical cancer prevention in females and HPV vaccination in males.

**Supplementary Information:**

The online version contains supplementary material available at 10.1007/s10096-023-04722-6.

## Introduction

At present, at least 15% of cancers worldwide are closely related to viral infections, and human papillomavirus (HPV) accounts for 5% of the global cancer burden [1, 2]. HPV is not only closely related to penile cancer, anal cancer, vaginal cancer, vulvar cancer, oropharyngeal cancer, head and neck squamous cell cancer and other cancers [3], but it has also been confirmed that persistent HPV infection is the principal cause of cervical cancer occurrence and development [4]. HPV is a sexually transmitted disease that is globally prevalent [5]. Almost 80% of people with active sexual life, whether males or females, have been infected with HPV [6]. A meta-analysis from china reported that the overall HPV prevalence of 15.6% in females and 14.5% in males. The risk of HPV infection in the two populations was at a similar level [7], So both males and females are susceptible to HPV infection. In recent years, most people have begun to pay attention to the impact of genital HPV infection status in heterosexual partners on females' cervical health. A cohort study from Tanzania found that the cervical HPV infection rate was extremely high after females' first sexual intercourse, and more than half of females were HPV positive within six months of their first sexual intercourse [8]. Several studies have evaluated HPV consistency among heterosexual partners, and a meta-analysis found that 25.5% (95% CI: 17.2–36.1%) of sexual partners were infected with one or more of the same HPV genotypes [9]. However, the current studies on genital HPV infection in heterosexual partners mainly focus on the prevalence, consistency and transmission dynamics of HPV genotypes in both sexes, while few studies focus on the impact of HPV positivity in males on HPV infection and cervical lesions in women.

From the perspective of regional sources of research, these surveys are mostly concentrated in Europe, America and Africa. Information on genital HPV infection in heterosexual partners or couples is limited in China. A questionnaire study in China found that only 22% of males had heard of HPV [10]. Due to the influence of traditional conservative concepts and the lack of relevant knowledge and education in China, males tend to have less awareness of the risk of HPV, which makes it more challenging to carry out these studies in China.

Therefore, the purpose of this study was to evaluate the prevalence and consistency of genital HPV infection in couples and to study the impact of HPV positivity status and infection type of husbands on cervical HPV infection and lesions in wives to better understand the burden of HPV between the sexes in Fujian, China, providing a basis for HPV and cancer screening programs in the Chinese population.

## Materials and methods

### Study population

This study was a longitudinal retrospective cohort study of genital HPV infection in couples from 2013 to 2021 conducted in the outpatient department of Fujian Maternal and Child Health Hospital. The survey population included married women with suspicion of underlying cervical disease. After obtaining consent from the couple, the outpatient physician performed the genital HPV-type test for the couple. From 2013 to 2021, a total of 283 couples were registered in the outpatient department. The inclusion criteria were that the couples had a similar baseline HPV detection time (interval ≤ 6 months) and had at least one valid HPV test result. Therefore, after exclusion according to the criteria, only 251 couples were eligible for the study. For the follow-up of females, we used the medical record card number of female patients to collect relevant examination results on the hospital's examination and pathology network system.

### Laboratory test

#### HPV testing

For females, clinicians used a sterile cytobrush to collect their cervical canal and external cervical canal and store exfoliated cells from the cervical transformation zone in a dedicated cell preservation solution. For males, urethral epithelial cells were collected using a moistened cotton swab, and exfoliated cells were also collected in preservation solution. Each sample was subjected to HPV type analysis by PCR-reverse dot blot using an HPV genotyping kit (Yaneng Biosciences (Shenzhen) Co., Ltd., China). The kit can detect 23 HPV genotypes, including 14 HR-HPV genotypes (HPV16, HPV18, HPV31, HPV33, HPV35, HPV39, HPV45, HPV51, HPV52, HPV56, HPV58, HPV59, HPV66, HPV68) and 9 non-HR-HPV genotypes (HPV6, HPV11, HPV42, HPV43, HPV53, HPV73, HPV81, HPV82, HPV83).

#### ThinPrep cytology testing (TCT)

The clinician uses a special cervical sampling brush to extend into the cervical canal, rotate the sampling brush in the same direction, and finally place the brush head in a vial containing cell fixative for rinsing, and then collect the exfoliated cells for thin-layer smear staining. All TCT smears were diagnosed by a professional pathologist under the microscope. Diagnose by condition: Negative for intraepithelial lesion or malignancy (NILM); Atypical squamous cells of undetermined significance (ASC-US); Atypical squamous cells cannot exclude high-grade squamous intraepithelial lesion (ASC-H); Low-grade squamous intraepithelial lesion (LSIL); High-grade squamous intraepithelial lesion (HSIL); Glandular epithelial cell abnormality.

#### Pathological biopsy

Colposcopy was performed on females with a positive TCT test result and/or high-risk HPV (HR-HPV) positivity. According to the results of the cervical acetic acid test and iodine solution staining, the images were evaluated, and a multipoint biopsy was carried out according to the abnormal part under the microscope. Tissue was sent for pathological examination. Diagnosis was based on colposcopy and histopathology: normal or chronic inflammation; mild dysplasia (cervical intraepithelial neoplasia I, CIN I); moderate dysplasia (CIN II); severe dysplasia, including carcinoma in situ (CIN III); carcinoma in situ and early infiltration; and infiltrating carcinoma.

### Related definitions

According to the evolutionary relationship of genes, HR-HPV can be divided into A9, A7 and A5/A6 groups. HPV A9 is homologous to HPV16 (including HPV16,31,33,35,52,58), HPV A7 is homologous to HPV18 (including HPV18,39,45,59,68), and group A5/6 includes HPV 51,56,66. In 251 couples, concordant infections are defined as couples who share at least one specific type of HPV type. Of the 251 females included in the study, only 140 had valid cervical biopsy results close to the HPV testing time (≤ 3 months). The pathological results of these participants were included in an ordered logistic regression using the following grading scales: Negative for Intraepithelial Lesion or Malignancy (NILM, *n* = 73), CIN I (*n* = 41), CIN II and CIN II above (*n* = 26). After follow-up, only 142 females had more than two records of cervical HPV genotyping. Risk of acquisition refers to the risk of HPV infection in different populations. The elimination of the virus was defined as two consecutive negative HPV tests.

### Statistics

Pearson’s chi-square test, Fisher's exact test, or Bonferroni's test were used to detect differences in HPV genotype frequencies between couples, and it was used to compare female HPV genotypes in different HPV infection statuses and infection modes of males. The Kappa consistency test was used to calculate the kappa values of HPV genotypes in couples. The kappa values were interpreted according to Fleiss guidelines and classified as follows: poor (kappa < 0.4), good (kappa = 0.4–0.75), and excellent (kappa > 0.75). Binomial logistic regression was used to analyze the risk of male HPV positivity status, on female HPV genotype infection and cervical lesions. The forest map and the Kaplan–Meier curve were created based on R 3.6.3, and the R package involved in visualization was the ggplot2 package. The other statistical analyses were carried out using the SPSS software version 26 software package (IBM, Chicago, USA). Differences at two-tailed *P* < 0.05 were considered statistically significant.

## Results

### Prevalence of different HPV genotypes among couples

As shown in Table 1, among 251 couples, the overall HPV infection rate was 42.2% for males and 78.1% for females. Among the HPV genotypes infecting males, HR-HPV accounted for the highest proportion (32.3%), and the most commonly detected HR-HPV genotypes were HPV 52(14.3%), HPV 51(5.6%), 16(4.8%), 58(4.4%), and the most commonly detected non-HR-HPV genotypes were HPV 43(5.6%), 42(5.2%), 53(4.8%). Compared with HPV A7 and A5/A6 in the alpha-papillomavirus species groups, the positive rate of HPV A9 infection was higher in males (22.7% vs 8.4%, 22.7% vs 8.8%). Among the HPV genotypes infecting females, HR-HPV also accounted for the highest proportion (66.5%), and the most commonly detected HR-HPV genotypes were HPV 16(17.9%), HPV 52(17.5%), 18(10.8%), 58(10.8%), and the most commonly detected non-HR-HPV genotypes were HPV 81(6.4%), 43(4.4%), 53(7.2%). Additionally, in the alpha-papillomavirus species groups, the number of females infected by the HPV-A9 group compared with the A7 and A5/A6 groups was higher (45.8% vs 19.5%, 45.8% vs 12.7%). There were no significant sex differences between partners in the risk of acquiring the specific HPV genotypes.

**Table 1:** Infection rates of different HPV genotypes in heterosexual couples

HPV genotypes	Male (*N* = 251)	Female (*N* = 251)	χ2	*P*-value*
Any HPV positive [%(n/N)] ^a^	42.2% (106/251)	78.1% (196/251)	67.321	< **0.001**
HR-HPV positive [%(n/N)] ^b^	32.3% (81/251)	66.5% (167/251)	58.941	< **0.001**
HPV 16 positive	4.8% (12/251)	17.9%(45/251)	21.552	< **0.001**
HPV 18 positive	1.6% (4/251)	10.8% (27/251)	18.188	< **0.001**
HPV 31 positive	0.8% (2/251)	2.4% (6/251)	1.143	0.285§
HPV 33 positive	1.6% (4/251)	3.6% (9/251)	1.974	0.160
HPV 35 positive	1.6% (4/251)	2.8% (7/251)	0.837	0.360
HPV 39 positive	2.0% (5/251)	2.8% (7/251)	0.341	0.559
HPV 45 positive	0.4% (1/251)	0.4% (1/251)	< 0.001	1.000§
HPV 51 positive	5.6% (14/251)	7.2% (18/251)	0.534	0.465
HPV 52 positive	14.3% (36/251)	17.5% (44/251)	0.952	0.329
HPV 56 positive	2.4% (6/251)	2.4% (6/251)	< 0.001	1.000
HPV 58 positive	4.4% (11/251)	10.8% (27/251)	7.289	**0.007**
HPV 59 positive	3.2% (8/251)	2.4% (6/251)	0.294	0.588
HPV 66 positive	2.8% (7/251)	3.6% (9/251)	0.258	0.611
HPV 68 positive	2.0% (5/251)	5.2% (13/251)	3.688	0.055
HPV A7 positive ^c^	8.4% (21/251)	19.5% (49/251)	13.015	< **0.001**
HPV A9 positive ^d^	22.7% (57/251)	45.8% (115/251)	29.752	< **0.001**
HPVA5/A6 positive ^e^	8.8% (22/251)	12.7% (32/251)	2.075	0.150
Non-HR-HPV positive[%(n/N)] ^f^	22.7% (57/251)	25.9% (65/251)	0.693	0.405
HPV 6 positive	2.0% (5/251)	2.0% (5/251)	< 0.001	1.000
HPV 11 positive	0.8% (2/251)	2.4% (6/251)	1.143	0.154§
HPV 42 positive	5.2% (13/251)	1.6% (4/251)	4.932	**0.026**
HPV 43 positive	5.6% (14/251)	4.4% (11/251)	0.379	0.538
HPV 53 positive	4.8% (12/251)	7.2% (18/251)	1.276	0.259
HPV 73 positive	0% (0/251)	0.4% (1/251)	< 0.001	1.000
HPV 81 positive	3.2% (8/251)	6.4% (16/251)	2.801	0.094
HPV 82 positive	0% (0/251)	0.8% (2/251)	0.502	0.479§
HPV 83 positive	1.2% (3/251)	0.8% (2/251)	< 0.001	1.000

^§^ is Yates's correction for continuity, the rest are all using Pearson’s χ2 test. Above df = 1

^*^ *P* < 0.05 indicates significant differences

a. Positive for any type of HPV

b. Positive for any of the genotypes 16,18,31,33,35,39,45,51,52,56,58,59,66,68 classified as HR-HPV

c. Positive for any of the genotypes 18,39,45,59,68 classified as HPV A7

d. Positive for any of the genotypes 16,31,33,35,52,58 classified as HPV A9

e. Positive for any of the genotypes 51,56,66 classified as HPV A5/A6

f. Positive for any of the genotypes 6,11,42,43,53,73,81,82,83 classified as non-HR-HPV

Statistically significant values are shown in bold

The infection rates of HPV 16, 18 and 58 in females were significantly higher than those in males (all *P* < 0.05). The infection rates of the A7 and A9 species were also significantly different between the two sexes (*P* < 0.001), but there was no significant difference in the A5/A6 species. The number of HPV 31, 33, and 68 infections in females was twice as high as that in males (2.4% vs 0.8%, 3.6% vs 1.6%, 5.2% vs 2.0%). Notably, among the 23 genotypes, only HPV42 infection in males was approximately three times higher than that in females (5.2% vs 1.6%). For the two highly carcinogenic genotypes of HPV16 and 18, the infection rate of HPV18 in males was three times lower than that of HPV16 (1.6% vs 4.8%), resulting in a much higher HPV18 positive ratio between males and females (10.8/1.6 vs 17.9/4.8).

### Consistency of different HPV genotypes in couples

Table S1 shows the comparative data between 251 couples with different HPV genotypes, which were classified as uniformly positive or discordant. Consistency was defined as both partners having the same HPV type. The overall agreement rate of HPV among heterosexual partners was 20.3% (17.5% for the high-risk type and 5.6% for the non-high-risk type). For any HR-HPV type, there was consistency between couples, but the overall consistency was poor (kappa = 0.163, *P* < 0.001). Among HR-HPV genotypes, HPV33, 51 and 58 showed good consistency (kappa = 0.593, 0.510, 0.403, respectively, all *P* < 0.001), while carcinogenic HPV16 and 18 showed weak consistency (kappa = 0.229 and 0.271, respectively, all *P* < 0.001). Among the different HR-HPV genotypes, the A5/A6 species showed good consistency (kappa = 0.550, *P* < 0.001), but the HPV A7 and A9 species showed poor consistency (kappa values were 0.312 and 0.279, respectively, all *P* < 0.001). Overall agreement for any non-HR-HPV type was general (kappa = 0.408, *P* < 0.001). Among non-HR-HPV genotypes, HPV53 showed good consistency (kappa = 0.541, *P* < 0.001).

### The influence of male HPV status and infection types on the females HPV genotypes infection

A total of 251 males were grouped by HPV infection status (Table 2). Among HPV-positive males, the overall prevalence of any HPV genotype in their wives was 81.1% (67.9% for HR genotypes and 32.1% for non-HR genotypes), and the most common HR-HPV genotypes were HPV52 (21.7%), 16 (13.2%) and 58 (13.2%). The prevalence of HPV A9 was the highest in the alpha-papillomavirus species groups, which was nearly twice as high as that in the A7 group (46.2% vs 24.5%) and more than twice as high as that in the A5/A6 group (46.2% vs 16.0%). Among the non-HR-HPV genotypes, the prevalence of HPV53 was the highest (10.4%).

**Table 2:** The influence of male HPV status on the distribution of HPV genotypes in females

Female HPV genotypes	Status of HPV infection in males		χ2	*p*-value*
	HPV positive (*N* = 106)	HPV negative (*N* = 145)		
Any HPV ^a^ [%(n/N)]	81.1% (86/106)	75.2% (109/145)	1.255	0.263
HR-HPV ^b^ [%(n/N)]	67.9% (72/106)	65.5% (95/145)	0.159	0.690
HPV 16	13.2% (14/106)	21.4% (31/145)	2.779	0.096
HPV 18	11.3% (12/106)	10.3% (15/145)	0.061	0.805
HPV 51	9.4% (10/106)	5.5% (8/145)	1.411	0.235
HPV 52	21.7% (23/106)	15.2% (22/145)	1.513	0.183
HPV 58	13.2% (14/106)	9.0% (13/145)	1.148	0.284
Other HR-HPV ^c^ genotypes	27.4% (29/106)	18.6% (27/145)	2.697	0.101
HPV A7^d^	24.5% (26/106)	15.9% (23/145)	2.927	0.087
HPV A9^e^	46.2% (49/106)	46.9% (68/145)	0.011	0.916
HPV A5/A6^f^	16.0% (17/106)	9.0% (13/145)	2.910	0.088
Non-HR-HPV ^g^ [%(n/N)]	32.1% (34/106)	15.2% (22/145)	7.563	**0.001**
HPV 42	0.9% (1/106)	2.1% (3/145)	0.037	0.847§
HPV 43	7.5% (8/106)	2.1% (3/145)	3.176	0.075§
HPV 53	10.4% (11/106)	4.8% (7/145)	2.833	0.092
HPV 81	6.6% (7/106)	6.2% (9/145)	0.016	0.899
Other Non-HR-HPV ^h^ genotypes	9.4% (10/106)	4.1% (6/145)	2.878	0.090

^§^ is Yates's correction for continuity, the rest are all using Pearson’s χ2 test. Above df = 1

^*^ *P* < 0.05 indicates significant differences

a. Positive for any type of HPV

b. Positive for any of the genotypes 16,18,31,33,35,39,45,51,52,56,58,59,66,68 classified as HR-HPV

c. Positive for any of the high-risk HPV genotypes excluding 16,18,51,52,58

d. Positive for any of the genotypes 18,39,45,59,68 classified as HPV A7

e. Positive for any of the genotypes 16,31,33,35,52,58 classified as HPV A9

f. Positive for any of the genotypes 51,56,66 classified as HPV A5/A6

g. Positive for any of the genotypes 6,11,42,43,53,81,73,82,83 classified as non-HR-HPV

h. Positive for any of the non-HR-HPV genotypes excluding 42,43,53

Statistically significant values are shown in bold

Partner-positive females had higher rates of HPV infection in most HR-HPV genotypes, including HPV51, 52, and 58, compared to partner-negative females. There was a significant difference in the non-HR-HPV infection rate between the two groups (32.1% vs 15.2%, *P* < 0.001). These data suggest that the genital HPV-positive status of husbands can increase their wives' risk of HPV infection, particularly non-HR-HPV genotypes.

HPV-positive males were divided into single infection (*n* = 70) and multiple infections (*n* = 36) according to the types of HPV infection, and the distribution of female HPV genotypes was observed (Table 3). In the case of single infection in males, the overall infection rate of female HPV was 78.6% (62.9% of HR genotypes and 24.3% of non-HR genotypes). In the case of multiple infections, the overall infection rate of female HPV of any genotype was 86.1% (77.8% of HR genotypes and 25.0% of non-HR genotypes). In the condition of multiple infections of males, the infection rate of almost all HPV genotypes in females was higher than that in the case of single infection of males, among which HPV51 (19.4% vs 4.3%, *P* = 0.029) and 52 (33.3% vs 15.7%, *P* = 0.037) in HR-HPV were significantly higher. The infection rates of the HPV A9 group (61.1% vs 38.6%, *P* = 0.028) and the A5/A6 group (38.9% vs 7.1%, *P* < 0.001) were also significantly different. Females had a higher infection rate of HPV18 when husbands had single HPV infection (12.9% vs 8.3%).

**Table 3:** The influence of male infection types on the distribution of HPV genotypes in females

Female HPV types	Types of HPV infection in males		χ2	*p*-value*
	Single infection ^i^ (*N* = 70)	Multiple infection ^j^ (*N* = 36)		
Any HPV ^a^ [%(n/N)]	78.6% (55/70)	86.1% (31/36)	0.883	0.347
HR-HPV ^b^ [%(n/N)]	62.9% (44/70)	77.8% (28/36)	2.429	0.119
HPV 16	11.4% (8/70)	16.7% (6/36)	0.204	0.652§
HPV 18	12.9% (9/70)	8.3% (3/36)	0.139	0.710§
HPV 51	4.3% (3/70)	19.4% (7/36)	4.743	**0.029**§
HPV 52	15.7% (11/70)	33.3% (12/36)	4.344	**0.037**
HPV 58	11.4% (8/70)	16.7% (6/36)	0.204	0.652§
Other HR-HPV genotypes^c^	18.6% (13/70)	66.7% (24/36)	24.202	< **0.001**
HPV A7^d^	22.9% (16/70)	30.6% (11/36)	0.742	0.389
HPV A9^e^	38.6% (27/70)	61.1% (22/36)	2.286	**0.028**
HPV A5/A6^f^	7.1% (5/70)	38.9% (14/36)	16.286	< **0.001**
Non-HR-HPV ^g^[%(n/N)]	24.3% (17/70)	25.0% (9/36)	0.007	0.935
HPV 42	1.4% (1/70)	0% (0/36)	0.835	0.361γ
HPV 43	4.3% (3/70)	13.9% (5/36)	1.917	0.166§
HPV 53	8.6% (6/70)	13.9% (5/36)	0.264	0.607§
HPV 81	7.1% (5/70)	5.6% (2/36)	< 0.001	1.000§
Other Non-HR-HPV^h^ genotypes	15.7% (12/70)	13.9% (5/36)	0.187	0.665

^§^ is Yates's correction for continuity, γ is Fisher's exact probability method, the rest are all using Pearson’s χ2 test. Above df = 1

^*^ *P* < 0.05 indicates significant differences

a. Positive for any type of HPV

b. Positive for any of the genotypes 16,18,31,33,35,39,45,51,52,56,58,59,66,68 classified as HR-HPV

c. Positive for any of the high-risk HPV genotypes excluding 16,18,51,52,58

d. Positive for any of the genotypes 18,39,45,59,68 classified as HPV A7

e. Positive for any of the genotypes 16,31,33,35,52,58 classified as HPV A9

f. Positive for any of the genotypes 51,56,66 classified as HPV A5/A6

g. Positive for any of the genotypes 6,11,42,43,53,81,73,82,83 classified as Non-HR-HPV

h. Positive for any of the Non-HR-HPV genotypes excluding 42,43,53

i. Infection with only one HPV genotype

j. Infection with two or more HPV genotypes

Statistically significant values are shown in bold

### Risk factors for HPV infection, HPV clearance and cervical lesion occurrence in females

The relationship between HPV positive status in males and different HPV genotypes in women is shown in Table 4.Compared with HPV-negative males, females with HPV-positive husbands were more likely to be infected with non-HR-HPV (OR = 2.634, *P* = 0.002). When males had non-HR-HPV and mixed HPV positivity, these conditions increased the risk of their partners infecting non-high-risk HPV (OR = 4.321 and 4.654, respectively, all *P* < 0.05). The male single infection increased the risk of non-HR-HPV infection in females compared with the reference population (OR = 2.354, *P* = 0.016). The multiple infection of males not only significantly increased the risk of non-HR-HPV infection in females (OR = 3.220, *P* = 0.006), which significantly increased the risk of HPV 52 and A7 species (OR = 2.502, 2.391, all *P* < 0.05).

**Table 4:** Influence of male HPV infection status and types on female HPV infection

Male HPV infection status	Females HPV infection (*N* = 251)											
	HR-HPV ^a^ positive		HPV 16 positive		HPV 52 positive		HPV A7 ^b^ positive		HPV A9 ^c^ positive		Non-HR- HPV ^d^ positive	
	OR (95%CI)	*P*-value*	OR (95%CI)	*P*-value	OR (95%CI)	*P*-value	OR (95%CI)	*P*-value	OR (95%CI)	*P*-value	OR (95%CI)	*P*-value
HPV negative	1.0	/	1.0	/	1.0	/	1.0	/	1.0	/	1.0	/
HPV positive	1.123 (0.658–1.915)	0.671	0.579 (0.290–1.157)	0.122	1.540 (0.796–2.979)	0.200	1.806 (0.967–3.374)	0.064	1.031 (0.623–1.708)	0.905	2.634 (1.429–4.853)	**0.002**
HR-HPV positive^e^	1.344 (0.685–2.637)	0.390	0.538 (0.221–1.308)	0.172	1.584 (0.720–3.486)	0.253	1.909 (0.910–4.004)	0.087	0.983 (0.529–1.826)	0.957	1.500 (0.685–3.284)	0.310
Non-HR-HPV positive^f^	0.806 (0.338–1.927)	0.628	0.717 (0.229–2.249)	0.569	1.452 (0.491–4.293)	0.500	1.304 (0.444–3.828)	0.629	0.756 (0.318–1.796)	0.527	4.321 (1.738–10.747)	**0.002**
Mixed-HPV positive^g^	1.075 (0.430–2.686)	0.877	0.538 (0.150–1.926)	0.341	1.529 (0.515–4.540)	0.445	2.148 (0.801–5.763)	0.129	1.588 (0.662–3.811)	0.300	4.654 (1.850–11.708)	**0.001**
Single infection^h^	0.876 (0.477–1.607)	0.669	0.484 (0.207–1.133)	0.094	1.063 (0.473–2.388)	0.882	1.546 (0.757–3.158)	0.232	0.802 (0.450–1.430)	0.455	2.354 (1.174–4.720)	**0.016**
Multiple infections^i^	1.766 (0.741–4.207)	0.199	0.801 (0.302–2.123)	0.656	2.502 (1.059–5.914)	**0.037**	2.391 (1.031–5.548)	**0.042**	1.701 (0.802–3.609)	0.166	3.220 (1.405–7.380)	**0.006**

Abbreviations: *CI* Confidence interval; *OR* Odds ratio

Binomial logistic regression is used to calculate Odds ratio. * *P* < 0.05 indicates significant differences

a. Positive for any of the genotypes 16.18.31.33.35.39.45.51.52.56.58.59.66.68 classified as HR-HPV

b. Positive for any of the genotypes 18.39.45.59.68 classified as HPV A7

c. Positive for any of the genotypes 16.31.33.35.52.58 classified as HPV A9

d. Positive for either type 6.11.42.43.53.81.73.82.83 classified as non-HR-HPV

e. Infection only with one or more genotypes of 16.18.31.33.35.39.45.51.52.56.58.59.66.68 is called HR-HPV

f. Infection only with one or more genotypes of 6.11.42.43.53.81.73.82.83 is called Non-HR-HPV

g. The type of co-infection in HR-HPV and Non-HR-HPV is called Mixed-HPV

h. Infection with only one HPV genotype

i. Infection with two or more HPV genotypes

Statistically significant values are shown in bold

**Fig. 1:**
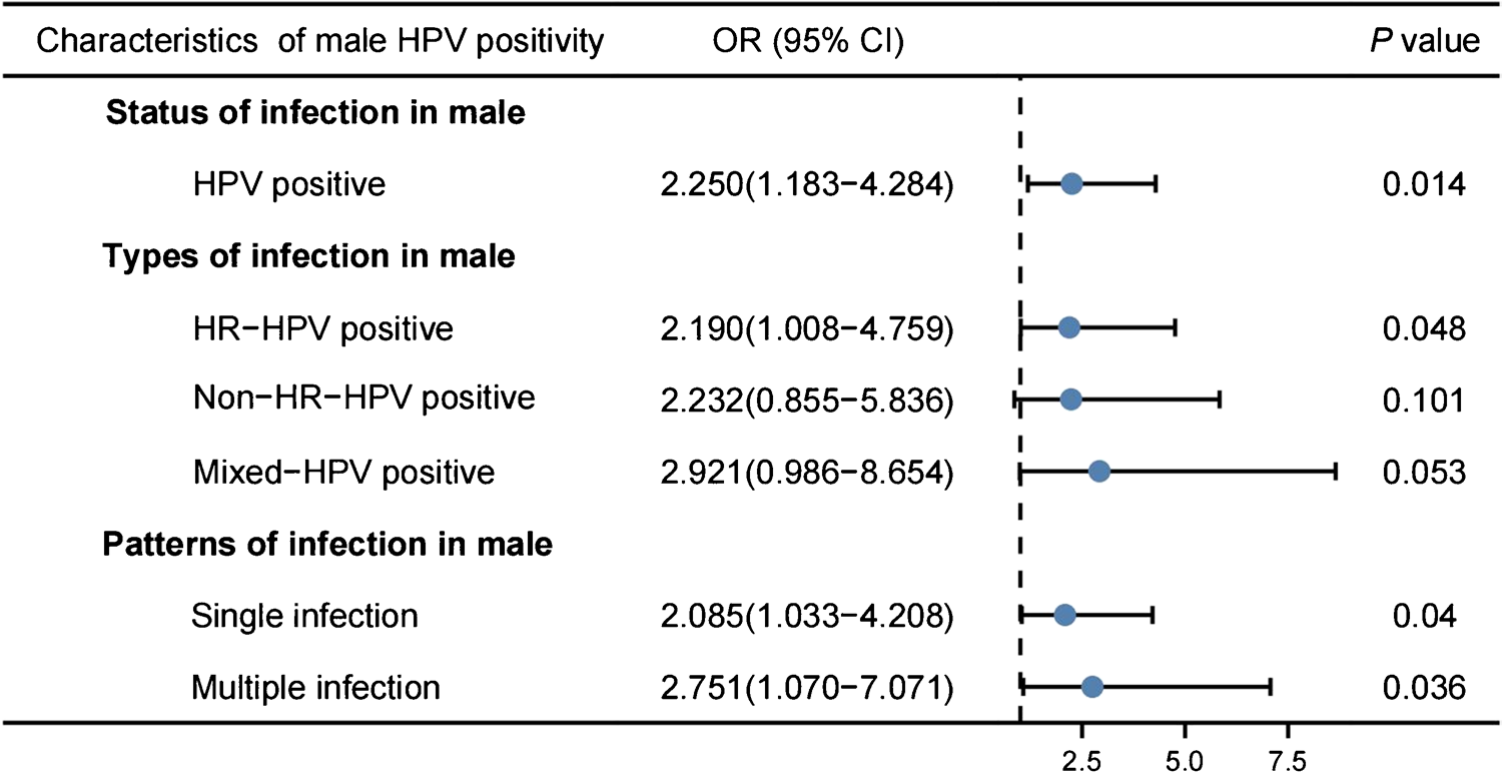
Kaplan-Meier curve of HPV clearance in HPV positive female, by pattern of HPV infection in male (N=142). (**A**) Results of HPV clearance in women with different infection status of male; (**B**) Results of HPV clearance in women with different infection patterns of male; (**C**) Results of HPV clearance in women with different infection types of male.

The mean duration of follow-up was 49.72 ± 4.16 months.The Kaplan–Meier curve showed (Fig. 1) that there was no significant change in HPV clearance time in females with HPV-positive husbands compared to females with HPV-negative husbands (Log rank *P* = 0.228). In the multi-group analysis of different infection status of males HPV, no factors were found to be associated with females HPV clearance (Log rank *P* = 0.416 and 0.683, respectively).

**Fig. 2:**
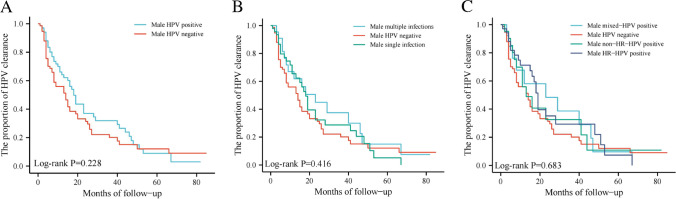
The influence of male HPV infection characteristics on the occurrence of cervical lesions in female (N=140). Abbreviations: OR, Odds ratio；CI, Confidence interval

A total of 140 females who were not treated with cervical conization and had valid biopsy results were selected, and the factors influencing the occurrence of cervical lesions in these females were analyzed by ordinal multiple logistic regression (Fig. 2). The results showed that compared with the females with HPV-negative partners, the likelihood of cervical lesions in females increased when the husbands were HPV-positive (OR = 2.250, *P* = 0.014), especially when the husbands were infected with HR-HPV, which is a risk factor for cervical health in females (OR = 2.190, *P* = 0.048). At the same time, both males HPV single-type or multiple-type infections were important indicators to promote the occurrence of cervical lesion in females (OR = 2.085, 2.751, all *P* < 0.05).

## Discussion

In this study, a retrospective study of 251 couples from Fujian, China was conducted to evaluate the differences and consistency of HPV type prevalence between couples and the correlation between genital HPV positivity in males and cervical HPV infection and lesions in females. Our data show no significant sex differences in the risk of acquisition for different genotypes of HPV in heterosexual couples, but the burden of HPV infection was higher in females than in males. The HPV-positive status of males had no effect on the clearance time of HPV infection in females. When males are in the state of non-HR-HPV and mixed infection of high-risk and non-high-risk genotypes, it will increase the burden of non-HR-HPV infection in females. HR-HPV positivity in males is a risk factor for the development of cervical lesions in females. Both single-type or multiple-type positivity in males will promote the risk of non-HR-HPV infection and the occurrence of cervical lesions in females.

There is little research on the prevalence of HPV among heterosexual couples in China. We reported the HPV infection burden in 251 couples, and the overall HPV infection rate in males was 42.2%, which was within the range of other studies on genital HPV infection rates in other countries around the world (1.3–72.9%) [11]. However, a meta-analysis of heterosexual males in China showed that the prevalence of HPV DNA in males was 11.3%-17.7% [12], which is much lower than our study results. This difference may be caused by different anatomical sampling sites and different HPV typing techniques. For females in our study, the infection rate of HPV of any type was as high as 78.1%, which was significantly higher than that in Northeast China (16.95%) [13], Northwest China (12.6%) [14], East China (32.2%) [15] and other regions. The overall prevalence rate of HPV in both sexes in our study was higher than that reported in previous studies. The possible reasons for this difference are not just related to the geographical location and economic development level but mostly due to the origin of the population in our study. The enrolled females were suspected of having cervical-related diseases and patients with cervical outpatient treatment. Therefore, the females HPV infection rate was high, which also increased the infection probability of their husbands. In the current study, heterosexual couples had the highest rates of HR-HPV infection, and no significant differences were observed in the common genital HPV infection genotypes between the sexes (males: 52, 51, 16, 58; females: 16, 52, 18, 58).

HPV A9 was the most common species in both sexes. These data were consistent with the common HPV infection genotypes in southern China [7, 16]. As a homologous genus of HPV 18, HPV A7 is associated with cervical adenocarcinoma. As a homologous species of HPV16, HPV A9 is associated with cervical squamous cell carcinoma.At present, this HPV grouping method is rarely used in clinic, but some studies have found that HPV A7 has more integration sites than A9, and the prognosis of cervical cancer caused by A7 is worse[17, 18].Although the integration rate of HPV A9 is lower than that of A7, the incidence of cervical cancer is higher [19, 20], so we still need to pay attention to the infection of different HPV groups in the population.

Although the infection type between husbands and wives may be similar, the research data show that females’ cervical HPV infection rates are significantly higher than those of males, which is consistent with several studies reporting results [7, 21] that may be related to the difference in characteristics of the female physiology structure compared with males. The higher temperature and moisture level in the vagina is a more conducive environment to the virus, making the rate of HPV infection clearance slower in females than in males. It is worth noting that a high incidence of males HPV42 has been reported in European countries such as Italy [22], Poland [23] and Switzerland [24]. However, in this study, only subtype HPV42 had a higher proportion of males than females, which may indicate that the clearance rate of male HPV42 is lower than that of other genotypes. However, the specific mechanism remains to be studied in the future. Lorenzon [25] demonstrated that male HPV type 42 positivity was a significant risk factor for female partner HPV infection. Guimera's study [26] also found that HPV42 was associated with typical anogenital squamous carcinoma. Therefore, although HPV42 is a low-risk type of HPV, males, as carriers of HPV transmission, have certain risks to the reproductive system health of sexual partners and themselves, which should be considered clinically.

In the present study, we observed that the overall agreement rate of HPV between couples was 20.3%, which was similar to the results of two studies from China (both results were 15.5%) [27, 28] but lower than the results of multiple foreign studies (results range 36.1%-68%) [9, 29–31]. The consistency results of different HPV genotypes did not meet our expectations, especially the oncogenic HPV16 and 18 genotypes, which showed weak consistency (kappa was 0.229 and 0.271, respectively). According to previous research reports, the consistency of HPV genotypes between sexual partners may be affected by the diversity of HPV genotypes examined, different sampling methods (especially male sampling), differences in the study population, sex concept education and other factors. Meanwhile, in this study, the number of cells sampled from male urethral epithelial exhumation cells was smaller than that of female cervical cells, and the lesion cells were easily missed, resulting in false negative results, which resulted in poor consistency of HPV genotypes in couples and was significantly different from other studies [27, 32, 33].

Our study hypothesizes that HPV positivity in husbands may be associated with cervical health in wives and that there may be some risk for infection in wives. Parada’s survey found that the main risk factor associated with female HPV DNA testing is the presence of HPV DNA in fixed sexual partners [34]. The Benevolent team's study has again confirmed that male HPV positivity is more common in HPV-positive female sexual partners. Males may be an important source of HPV transmission between sexual partners [35]. Therefore, our study first compared the infection situation of two groups of females whose husbands were HPV positive or negative. The data found that for most HR-HPV genotypes, the infection rate of females whose husbands were HPV positive was higher. The results are consistent with what we would expect, but the only interesting thing is that HPV16 has a higher infection rate in females with HPV-negative husbands. Feixue Wei's study found that 42% of females sustained type 16 infection after 6 months of follow-up, while HPV 16 infection did not persist in males, and the results were similar after 12 months of follow-up [36]. This might explain the existence of our data.

Subsequently, we continued to analyze the male HPV positivity status and the infection types.The correlation analysis was carried out on the female HPV infection. It was found that when husbands were HPV positive, especially with HR-HPV positivity or non-high-risk single positivity status, females were more likely to have a non-HR-HPV infection. Until now, HPV research has mainly focused on the HR-HPV virus itself and its pathogenic mechanism, and less attention has been given to non-HR-HPV genotypes, which cause more benign diseases such as genital warts. However, a recent study by a team from the National Institute of Research in the United States highlighted that infection with some non-high-risk HPV genotypes can significantly increase the risk of cervical precancerous lesions in people who have been vaccinated with bivalent HPV, resulting in an increased risk of cervical cancer [37]. Therefore, these results may have certain reference values. For females with HPV-positive husbands, it is urgent and important to vaccinate with more coverage of multivalent HPV vaccine to prevent the occurrence and development of cervical lesions. In our study, it was also reported that various positive HR-HPV status in males were significant risk factors for female cervical lesions. Irrelevant to whether the male partner was infected with a single-type or multiple-type infection, it would increase the burden of female cervical lesions. These results are also consistent with the conclusions of previous studies [38]. The sexual partners of females with high-grade cervical lesions are important hosts and vectors of HPV infection, so males have the risk of maintaining virus transmission, leading to the recurrence of cervical diseases in females. In this regard, we must take timely preventive treatment measures for HPV-infected heterosexual couples to avoid the possibility of disease.

To the best of our knowledge, this is the first study that evaluates the presence of HPV positivity status and types in the husbands as the variable associated with genital HPV clearance in wives. We observed that HPV-positive males were not associated with female HPV clearance. A study found that the absence of multiple sexual partners during female HPV infection was associated with early HPV clearance (AHR, 5.52 [95% CI, 3.28–9.30]) and that having more sexual partners prolonged HPV infection in females [39]. Our study population targeted stable, monogamous couples, so the selection of this population may be related to the emergence of the results of this study.

There are some limitations to our study. First, we only recruited females who visited the outpatient clinic and their husbands who had marital relationships. However, no follow-up was conducted on females' previous history of cervical conization, marital baseline information and living habits (including sexual behaviors), which may lead to bias in the final analysis. Furthermore, our study was retrospective, leading to many people being lost to follow-up. There is a possibility that the infection was only a transient infection. Persistent and transient infections were not separated, leading to the impact of incidence, consistency and other data in the study. Finally, our understanding of the males sampling sites is too limited, the urethra has the lowest detection rate of HPV in many genital anatomy sites, including the scrotum, corona of the glans penis, and anus [40]. Therefore, this may cause us to underestimate the incidence and consistency of HPV in males, thus affecting the analysis of the correlation between HPV infection in males and females. Fourth, the bivalent HPV vaccine has been approved for marketing in China since 2016, but the current coverage rate of HPV vaccine in China is still very low.According to statistics, by 2020, the cumulative full coverage rate of HPV vaccine for women aged 9 to 45 in China is only 2.24%[41].Studies have shown that HPV vaccine is effective against both persistent HPV-associated infections and genital precancerous lesions[42, 43], so our study did not collect HPV vaccination history in females, which may affect our analysis.

## Conclusion

In conclusion, our results show that HPV52, 16 and 58 are the common HPV types in couples in the Fujian region. Except for type 42, the infection rate of females HPV were higher than that of males. Males HPV positivity increases the burden of non-HR-HPV infection and increases the observed frequency of HR types.It also increases the risk of cervical lesions developing in females. The presence of HPV in males was not associated with the clearance time of HPV infection in females. The role of males in the occurrence and development of cervical cancer in females should not be ignored. This information has good public health significance. It is hoped to provide a reference value for cervical cancer prevention in females and HPV vaccination in males.

### Supplementary Information

Below is the link to the electronic supplementary material.

Supplementary file1 (DOCX 17 KB)

## Data Availability

The datasets analysed during the current study are not publicly available, but are available from the corresponding author on reasonable request.
