# Supplementary material for: HPV positivity status in males is related to the acquisition of HPV infection in females in heterosexual couples
Source: Eur J Clin Microbiol Infect Dis. 2024 Jan 4;43(3):469–80. doi: 10.1007/s10096-023-04722-6 (PMC10917843; doi:10.1007/s10096-023-04722-6)
Supplement: Supplementary file 1 — Supplementary file1 (DOCX 17 KB) [file 10096_2023_4722_MOESM1_ESM.docx]

**Table S1****. Concordance of human papillomavirus genotype infection in heterosexual couples ^a^.**

| **HPV type** | **Male/Female** | | | **Concordance**  **[% n(+/+)/N]** | **Kappa(95%CI)** | **P-value*** |
| --- | --- | --- | --- | --- | --- | --- |
|  | **n (+/+)** | **n (+/-)** | **n (-/+)** |  |  |  |
| Any HPV ^b^[%（n/N）] | 51 | 20 | 109 | 20.3% | 0.082  (-0.010-0.174) | 0.094 |
| HR-HPV ^c^[%（n/N）] | 44 | 16 | 95 | 17.5% | 0.163  (0.067-0.259) | **0.001** |
| HPV 16 | 7 | 2 | 35 | 2.8% | 0.229  (0.076-0.382) | **＜0.001** |
| HPV 18 | 3 | 0 | 15 | 1.2% | 0.271  (0.026-0.516) | **＜0.001** |
| HPV 33 | 3 | 0 | 4 | 1.2% | 0.593  (0.232-0.954) | **＜0.001** |
| HPV 51 | 5 | 1 | 8 | 2.0% | 0.510  (0.234-0.786) | **＜0.001** |
| HPV 52 | 16 | 10 | 26 | 6.4% | 0.393  (0.234-0.552) | **＜0.001** |
| HPV 58 | 7 | 4 | 14 | 2.8% | 0.403  (0.183-0.623) | **＜0.001** |
| HPV A7^d^ | 7 | 2 | 24 | 2.8% | 0.312  (0.126-0.498) | **＜0.001** |
| HPV A9^e^ | 33 | 11 | 68 | 13.1% | 0.279  (0.169-0.389) | **＜0.001** |
| HPV A5/A6^f^ | 12 | 4 | 13 | 4.8% | 0.550  (0.362-0.738) | **＜0.001** |
| Non-HR-HPV ^g^[%(n/N)] | 14 | 9 | 22 | 5.6% | 0.408  (0.239-0.577) | **＜0.001** |
| HPV 53 | 5 | 1 | 7 | 2.0% | 0.541  (0.261-0.821) | **＜0.001** |
| HPV 81 | 3 | 2 | 9 | 1.2% | 0.334  (0.040-0.628) | **＜0.001** |

**Abbreviations:** CI, confidence interval.

**NOTE:**

Cohen's Kappa is used to calculate kappa value. * P<0.05 indicates significant differences.

a. Concordance refers to the presence of a common HPV genotype between husband and wife.

b. Positive for any type of HPV.

c. Positive for any of the types 16,18,31,33,35,39,45,51,52,56,58,59,66,68 classified as HR-HPV.

d. Positive for any of the types 18,39,45,59,68 classified as HPV A7.

e. Positive for any of the types 16,31,33,35,52,58 classified as HPV A9.

f. Positive for any of the types 51,56,66 classified as HPV A5/A6.

g. Positive for any of the types 6,11,42,43,53,81,73,82,83 classified as Non-HR-HPV.

**Article title:** The infection of HPV in males is related to the acquisition of HPV events in females in heterosexual couples

**Journal name:** European Journal of Clinical Microbiology & Infectious Diseases

**Author names:** Yuxuan Huang#, Yafang Kang#, Ye Li, Liangzhi Cai, Qibin Wu, Dabin Liu, Xiaodan Mao, Leyi Huang, Kelvin Stefan Osafo, Yan Zhang, Shuxia Xu, Binhua Dong*, Pengming Sun*

**Corresponding authors:**

Pengming Sun, M.D., Ph.D., Professor, Fujian Maternity and Child Health Hospital, College of Clinical Medicine for Obstetrics & Gynecology and Pediatrics, Fujian Medical University, 18 Daoshan Road, Fuzhou 350001, Fujian, P.R. China (Phone: +86-591-87558732; Fax: +86-591-87551247; E-mail: sunfemy@hotmail.com; fmsun1975@fjmu.edu.cn)

Binhua Dong, M.D., Fujian Maternity and Child Health Hospital, College of Clinical Medicine for Obstetrics & Gynecology and Pediatrics, Fujian Medical University, 18 Daoshan Road, Fuzhou 350001, Fujian, P.R. China (Phone: +86591-87558732; Fax: +86-591-87551247; E-mail: dbh18-jy@126.com; dongbinhua86@fjmu.edu.cn)
